# An altered gut microbiome in pre-eclampsia: cause or consequence

**DOI:** 10.3389/fcimb.2024.1352267

**Published:** 2024-05-07

**Authors:** Clara Deady, Fergus P. McCarthy, Aaron Barron, Cathal M. McCarthy, Gerard W. O’Keeffe, Siobhain M. O’Mahony

**Affiliations:** ^1^ Department of Anatomy and Neuroscience, University College Cork, Cork, Ireland; ^2^ APC Microbiome Ireland, University College Cork, Cork, Ireland; ^3^ Department of Obstetrics and Gynecology, University College Cork, Cork, Ireland; ^4^ The Infant Research Centre, University College Cork, Cork, Ireland; ^5^ Department of Pharmacology and Therapeutics, University College Cork, Cork, Ireland; ^6^ Cork Neuroscience Centre, University College Cork, Cork, Ireland

**Keywords:** pre-eclampia, microbiome, inflammation, probiotics, diet-intervention

## Abstract

Hypertensive disorders of pregnancy, including pre-eclampsia, are a leading cause of serious and debilitating complications that affect both the mother and the fetus. Despite the occurrence and the health implications of these disorders there is still relatively limited evidence on the molecular underpinnings of the pathophysiology. An area that has come to the fore with regard to its influence on health and disease is the microbiome. While there are several microbiome niches on and within the body, the distal end of the gut harbors the largest of these impacting on many different systems of the body including the central nervous system, the immune system, and the reproductive system. While the role of the microbiome in hypertensive disorders, including pre-eclampsia, has not been fully elucidated some studies have indicated that several of the symptoms of these disorders are linked to an altered gut microbiome. In this review, we examine both pre-eclampsia and microbiome literature to summarize the current knowledge on whether the microbiome drives the symptoms of pre-eclampsia or if the aberrant microbiome is a consequence of this condition. Despite the paucity of studies, obvious gut microbiome changes have been noted in women with pre-eclampsia and the individual symptoms associated with the condition. Yet further research is required to fully elucidate the role of the microbiome and the significance it plays in the development of the symptoms. Regardless of this, the literature highlights the potential for a microbiome targeted intervention such as dietary changes or prebiotic and probiotics to reduce the impact of some aspects of these disorders.

## 1 Introduction

Hypertensive disorders of pregnancy are among the most common complications of pregnancy, affecting approximately between 5-10% of all pregnancies globally (Hutcheon et al., 2011). These can lead to a number of serious and debilitating complications that affect both the mother and the fetus (Chappell et al., 2021). The World Health Organization has reported that hypertensive disorders of pregnancy account for 14% of maternal deaths in developed countries, which increases by up to 22% in the Caribbean and parts of Latin America, making it the leading cause of maternal mortality (Khan et al., 2006).

Hypertensive disorders of pregnancy are an umbrella term for multiple disorders, including gestational hypertension, chronic hypertension, and pre-eclampsia (PE), which has the highest incidence rate, impacting 3-5% of global pregnancies (Mol et al., 2016). PE is clinically diagnosed as *de novo* hypertension (≥140 mmHg systolic and/or ≥90 mmHg diastolic) after the 20^th^ week of gestation in addition to one of the following: 1) proteinuria (≥300 mg of protein in 24 hours); 2) uteroplacental dysfunction (fetal growth restriction); 3) maternal organ dysfunction, including neurological complications (such as hyperreflexia, changes in cognitive function, visual disturbances, or stroke), liver involvement (such as epigastric or right upper abdominal pain, increased transaminases levels), renal insufficiency (increased creatinine levels), and hematological issues (such as hemolysis or thrombocytopenia) (Hypertension in pregnancy: executive summary, 2013; Tranquilli et al., 2014).

Despite the potential consequences of PE on the mother and the fetus, there is currently limited evidence regarding the molecular drivers of the pathophysiology of PE. However, the predominant theory proposes defective placentation (Magley and Hinson, 2022). Ultimately, this leads to a reduced uteroplacental blood flow, resulting in placental ischemia and oxidative stress, which modifies the arteries from low-flow, high-resistance to high-flow, low-resistance vessels (Magley and Hinson, 2022). The continuous cycle of ischemia and reperfusion leads to the release of free radical species, pro-inflammatory cytokines, and antiangiogenic factors (Makris et al., 2007; Uzan et al., 2011). Endothelial dysfunction is also present due to improper placentation (Opichka et al., 2021). Women with PE tend to have altered inflammatory profiles, in particular increased levels of circulating pro-inflammatory cytokines, including tumor necrosis factor-α, C-reactive protein, and interleukin-6 (IL-6) (Teran et al., 2001; Xiao et al., 2012). There is currently no cure for PE with the only effective treatment being delivery of the baby in addition to the clinical management of maternal hypertension with antihypertensives (Amaral et al., 2017).

**Figure 1:**
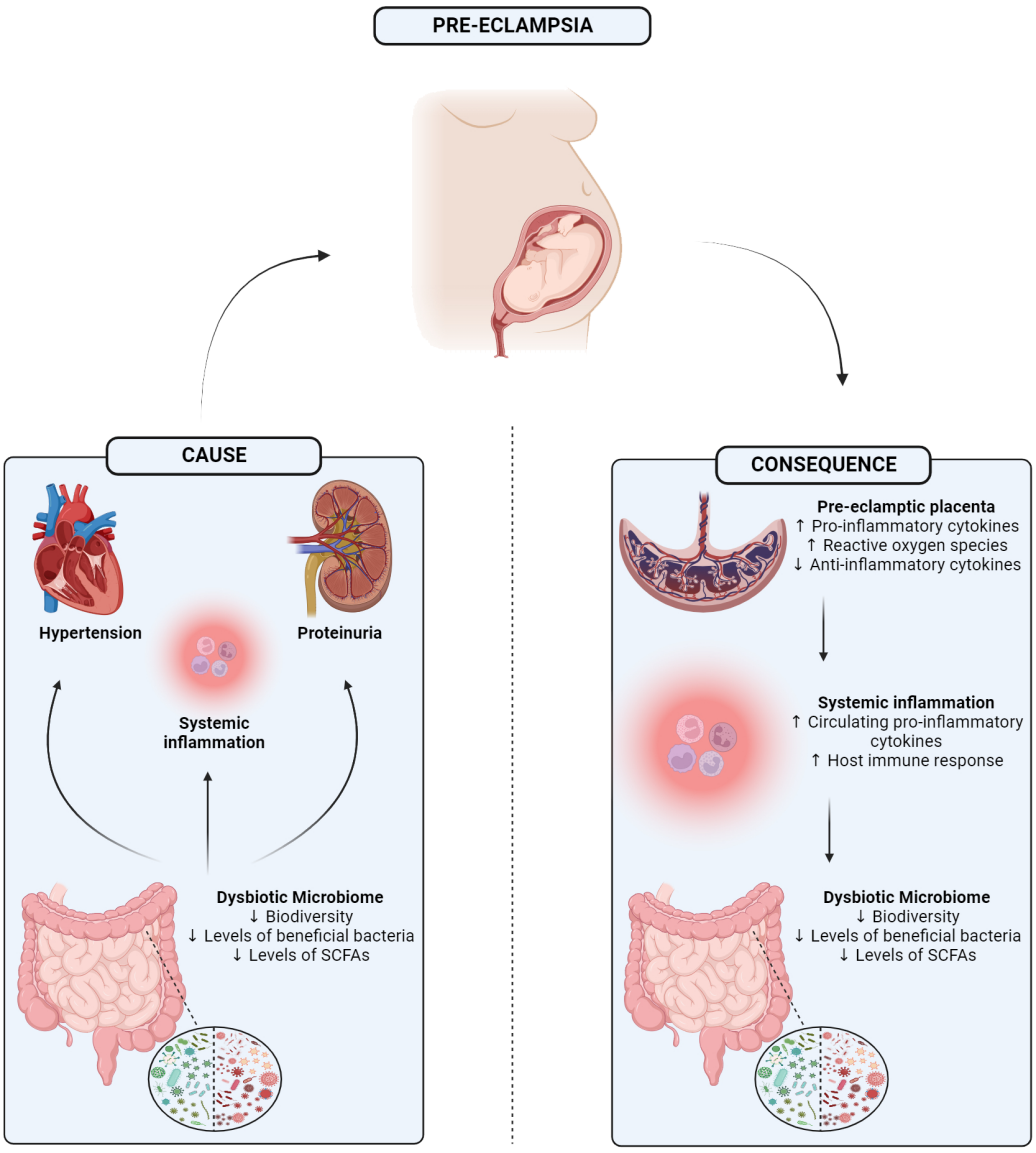
Graphical abstract showing the 1) potential mechanism in which a dysbiotic microbiome may be driving 3 of the main symptoms of pre-eclampsia, or 2) how separately pre-eclampsia itself may trigger a dysbiotic gut microbiome potentially mediated through the placenta. SCFA, Short-chain fatty acid. Created with BioRender.com.

There is now substantial pre-clinical and clinical evidence that the microbiome, particularly that of the distal end of the gastrointestinal tract, plays an important role in the pathogenesis of diseases (O’Mahony et al., 2017). More recently, symptoms of PE have also been associated with an aberrant microbiome (Jin et al., 2022). Currently, it is unknown if a disrupted microbiome is an instigator of the onset of PE and associated symptoms or a consequence of the disorder itself. It may be the case that both of these scenarios are possible. Yet, despite this uncertainty, there are notable and impactful alterations in the various microbiome niches during pregnancy that may potentially contribute to this condition (**Figure 1**).

## 2 The microbiome

From an evolutionary perspective, the human body has developed alongside a strong microbial influence, and this has led to various microbial niches forming in various parts of the body, such as the oral cavity, the urogenital tract, and perhaps most importantly, the gastrointestinal (GI) tract (Cryan et al., 2019). These areas harbor a significant wealth and diversity of microorganisms that live symbiotically. It is now known that microbial cells outnumber human cells 1.3:1 in the body (Sender et al., 2016). The term microbiome refers to the microorganisms and the products they produce, as well as their genetic profile. The GI tract alone may have up to 1000 bacterial species that come from 50 different phyla (Qin et al., 2010; Adak and Khan, 2019). While some of the bacteria that reside in the GI tract may be harmful, many are in fact beneficial. Genera such as Bifidobacteria, amongst others, have been shown to reduce inflammation, modulate the immune response, and produce essential vitamins (LeBlanc et al., 2013). Other genera such as Lactobacillus may have a beneficial effect on various conditions such as irritable bowel syndrome, stress and anxiety, and has even been shown to improve cognition in elderly patients (Diop et al., 2008; Chung et al., 2014; Chong et al., 2019). Due to the numerous and diverse benefits associated with these genera, they are commonly consumed as probiotics, wherein live bacteria taken in large volumes exert a positive influence on the host (Butel, 2014).

While an “optimal microbiome” has not been defined, considering the influence of numerous intrinsic and environmental factors, a healthy state has previously been characterized as the absence of any disease (Aagaard et al., 2013). The microbiome changes dramatically as the host ages, while also being influenced by host genetics and sex (Haro et al., 2016b; Badal et al., 2020; Caputi et al., 2022; Diviccaro et al., 2022). Also, it is commonly appreciated that the microbiome is readily impacted by lifestyle factors such as diet and exercise (Sandhu et al., 2017; Zmora et al., 2019; Berding et al., 2021). Continuous consumption of fatty foods, antibiotics, and a sedentary lifestyle, amongst others, have a detrimental effect on the microbiome, overall health, and subsequently one’s mood, and mental wellbeing (Dalton et al., 2019; Hao et al., 2020; Berding et al., 2021). Stress during all stages of life impacts negatively on the physiology of the gut as well as the microbiome with increased permeability, local inflammation, decreased diversity and reduced beneficial microbes (Karl et al., 2018; Malesza et al., 2021). Furthermore, stress *in utero* and in the postnatal period have been linked to sub-optimal microbiome colonization and neurodevelopment of the baby (Galley et al., 2023). Conversely, it has been shown that a healthy diet and subsequently, an optimal microbiome during pregnancy positively impacts the long-term development of the child (Jacka, 2017).

The gut microbiome has been shown to impact both brain and behavior through the bidirectional communication of the gut-brain axis (Cryan et al., 2019). Through the enteric and autonomic nervous systems, along with the immune and endocrine systems, the gut and the brain are able to transfer information and modulate each other (Cryan et al., 2019). Some of the mechanisms involved are through the neurotransmitters and neuropeptides that are either produced by the microorganisms that reside in the gut or metabolized by them and subsequently affect the brain. Molecules such as histidine, glutamate, and tryptophan can be converted into histamine, gamma-aminobutyric acid, and serotonin respectively by bacteria presiding in the gut, and these major neurotransmitters play a key role in mood, sleep, and overall behavior (Cryan et al., 2019). Metabolites, which are the products of bacteria, play a crucial role in aiding in the communication between the two end organs also. Short-chain fatty acids (SCFAs) such as butyrate and acetate, produced from the fermentation of dietary fiber, have suggested roles ranging from aiding in neuroimmune function, to supporting the blood brain barrier (Soliman et al., 2012; Braniste et al., 2014; Pascale et al., 2018; Silva et al., 2020).

Through extensive studies, a strong link has been found between the gut microbiome and its role in the health and disease of the host. As previously described, beneficial bacteria not only allow for the optimal maintenance of the GI tract, but also can help ameliorate symptoms in disorders of the GI tract as well as external to the gut and even central nervous system disorders. However, the converse is also true. Whether they are a cause or consequence, significant microbial alterations have been found in various disease states. This includes neurodegenerative disorders such as multiple sclerosis, where dysbiosis was evident and symptoms correlated with an increase in *Akkermansia muciniphila*, along with *Acinetobacter calcoaceticus* (Cekanaviciute et al., 2017). These changes also extend to brain injuries such as stroke, where multiple commensal bacterial species have been greatly depleted, and the abundance of pathogens is increased (Yin et al., ). It has been suggested that the gut microbiome may even have an influence over the levels of neuroinflammation post-stroke via T-cell trafficking (Benakis et al., 2016). Drastic changes in the gut microbiome have also been seen in those suffering from depression and schizophrenia (Naseribafrouei et al., 2014; Castro-Nallar et al., 2015; Jiang et al., 2015). Building on this, changes have been noted in the levels of SCFAs in neurological conditions, from autism spectrum disorder to Alzheimer’s disease (Wang et al., 2012; Zhang et al., 2017).

## 3 The gut microbiome during pregnancy

### 3.1 Normal gut microbiome changes seen in pregnancy

During pregnancy, significant changes occur in the female body to allow for the rapid growth of the fetus, as well as to support and nourish. The maternal gut microbiome is one of these systems that is forced to adapt. Throughout pregnancy, the composition of the gut microbiome is influenced by the endocrine system, the immune system, and the metabolic changes regularly seen (Neuman and Koren, 2017). While hormones such as estrogen and progesterone have not been directly shown to alter the microbiome, previous work has demonstrated that hormones can influence microbial growth, and that these microbes respond to them appropriately (Neuman et al., 2015).

In relation to the immune system, severe changes occur to prevent fetal rejection, amongst other necessary functions. This immune modulation is thought to impact the microbiome composition in some way (Neuman and Koren, 2017). Furthermore, during implantation of the embryo, there are high levels of inflammation, seen in the intestinal mucosa (Mor and Cardenas, 2010; Koren et al., 2012). This high level of inflammation soon decreases during mid-pregnancy. However, as the pregnancy progresses, the environment shifts towards a more pro-inflammatory state once again to prepare for delivery (Mor and Cardenas, 2010). Higher levels of Actinobacteria and Proteobacteria are typically found due to these high levels of inflammation, which serve a purpose of protecting the fetus, along with lower levels of the anti-inflammatory Firmicutes and Clostridiales (Gorczyca et al., 2022; Ionescu et al., 2022).

The microbiome of a healthy, pregnant woman has been compared to that of someone suffering from metabolic syndrome (Nuriel-Ohayon et al., 2016). This includes symptoms such as insulin resistance, weight gain, glucose intolerance, and continuous inflammation (Nuriel-Ohayon et al., 2016). This is coupled with a reduction in α-diversity and increased β-diversity (Koren et al., 2012). The similarities between pregnancy and metabolic syndrome have been shown via faecal microbial transfer from women in the third trimester to mice where they display insulin resistance, weight gain, and a pro-inflammatory phenotype (Koren et al., 2012). Some of the same microbial signatures can also be seen, with levels of SCFA-producing Faecalibacterium reduced, as is in metabolic syndrome (Haro et al., 2016a). In general, the levels of SCFAs such as butyric and propionic acid, along with acetic acid, which has been shown to be the most dominant (Hayward et al., 2020; Nilsen et al., 2020; Ziętek et al., 2021).

Other notable changes include an increased level of Firmicutes, Bifidobacterium, and the mucin-digesting Akkermansia, and this is strongly associated with the growing need for energy extraction and storage (Gorczyca et al., 2022; Ionescu et al., 2022). Most of the alterations are seen in the later stages of pregnancy (Neuman and Koren, 2017). It is important to note that the overall composition of the maternal gut microbiome is also strongly influenced by multiple external factors, such as pre-conception and current diet, along with any medications such as antibiotics (Gohir et al., 2015; Khan et al., 2016). All of these changes together would be classified as serious dysbiosis in a non-pregnant individual but are essential during pregnancy to support the fetus as it develops.

The maternal gut microbiome seems to also play a role in placental growth and development. This has been shown via animal models with a depleted microbiome, where placental growth was found to be stunted and significantly reduced (Pronovost et al., 2023). This finding coincided with reduced volume and density at the site of maternal-fetal exchange, termed the placental labyrinth, which subsequently reduced fetal-placental vasculature (Pronovost et al., 2023). Several further animal studies have demonstrated the positive effects that SCFA supplementation has on the developing placenta, including increased placental size, improved spiral arteries, and reduced inflammatory markers (Jin et al., 2022; Pronovost et al., 2023). This highlights the potential need for appropriate levels of SCFAs, such as butyrate and proprionate, to be maintained across the gestational period.

While the gut microbiome seems to play an important role in supporting the placenta, as of recent, more studies have examined the potential of a placental microbiome. However, this has proved to be quite controversial. There are stark differences in published findings, some strongly arguing for the presence of microbes in the placenta, while other studies reveal nothing but sterility (Aagaard et al., 2014; Pelzer et al., 2017; Perez-Muñoz et al., 2017; Kennedy et al., 2023). However, if one is to assume that the placental microbiome does in fact exist, it is unknown how it forms. Current theories suggest the translocation of bacteria from the gut microbiome. This transfer from the gut has previously been shown in pregnant mice, where the bacteria were then found to populate the mammary glands and certain lymph nodes (Perez et al., 2007). Knowing this, it has been suggested that there may be hematogenous transfer of microbes from the epithelial gaps of the lumen of the gut to the placenta, and they begin to colonize here (Walker et al., 2017). However, this cannot be confirmed as of now.

The importance of the maternal microbiome is evident well beyond pregnancy. There is strong evidence that demonstrates the importance of a healthy microbiome for fetal development and predisposition to certain disorders. This has been shown for various neuropsychiatric and cardiometabolic disorders (Basak et al., 2022). One of the most notable set of findings comes from mice without a microbiome, known as germ free, which found that the impacted offspring had an altered serotonergic system and levels of brain-derived neurotrophic factor, neurogenesis in the postnatal period, myelination, and that the function of areas such as the hippocampus and amygdala is also changed, areas involved in autism spectrum disorder and other neuropsychiatric conditions (Basak et al., 2022). In 2020, a large study was published that linked depletion of the maternal gut with deficient thalamocortical axons (Vuong et al., 2020). This structural change led to deficits in offspring tactile sensitivity, which was then successfully rescued following administration of certain microbial metabolites (Vuong et al., 2020). All these *in vivo* studies highlight how crucial a healthy, optimal gut microbiome is for correct neurodevelopment, and how detrimental the consequences may be to the affected offspring.

### 3.2 Changes seen in the gut microbiome of pregnant women with pre-eclampsia

Numerous studies have found drastic and significant shifts in the gut microbiome of women with PE in comparison to women with healthy pregnancies. A study published in 2021 found significant decreases in Varibaculum, Prevotella, Lactobacillus, and Porphyromonas genera when compared to normotensive pregnancies, coupled with an increase in Bacteroidetes (Huang et al., 2021). In particular, Prevotella, which is known to aid in the protection against pathogens and produces SCFAs, was reduced by 50% in PE in comparison to healthy pregnant women (Kovatcheva-Datchary et al., 2015; Huang et al., 2021).

Studies have also found that women with PE have lower levels of Coprococcus, which is a known producer of the important and beneficial SCFA, butyrate (Altemani et al., 2021). Interestingly, lower levels of butyrate are also seen before the onset of the PE symptoms, and this has even been suggested to be involved in increasing the risk of developing PE, highlighting the potential role of the gut microbiome in the development of PE (Altemani et al., 2021). Furthermore, it has been shown that PE leads to an increased concentration of circulating lipopolysaccharide (LPS), which is known to be associated with inflammatory processes, along with Gammaproteobacteria and Enterobacteriaceae (Kell and Kenny, 2016; Wang et al., 2019). The differences between the microbiome of normotensive and PE pregnancies has also been investigated in relation to gestational age, and while no differences were found during the 2^nd^ trimester, a significant shift was seen in the third trimester when compared to normotensive pregnancies (Wang et al., 2020). The finding showed an increase in Bacteroidetes and Proteobacteria, mirroring findings from other studies, and a decrease in Firmicutes, which, as previously discussed, is vital for energy harvesting (Wang et al., 2020).

Building on this, it has been found that certain gut microbiome deficits can be linked to the clinical symptoms of PE. *Akkermansia muciniphila*, a beneficial microbe that produces many metabolites from degrading intestinal mucus, such as SCFAs, has been associated with metabolic health amongst other things (Zhang et al., 2019). Studies have found reduced Akkermansia in the intestines of women with PE, and this was negatively correlated with hypertension and proteinuria levels (Jin et al., 2022). These women displayed increased inflammation and significantly impaired barrier function, reduced levels of propionic and butyric acid in the faeces and placenta of women with PE, and the authors suggest that it may be through this that PE may develop (Jin et al., 2022). Furthermore, they note the potential of incorporating the maternal gut profile to aid in improving the diagnostic accuracy of PE (Jin et al., 2022). Administration of Akkermansia and both butyrate and proprionate to PE rats improved not only the clinical symptom of hypertension, but gut barrier function was improved, placental inflammatory factors were downregulated at both the mRNA and protein level, and improved spiral artery remodeling was noted (Jin et al., 2022). Other faecal microbial transplant studies have also reported similar results of microbiome alterations, poor barrier function, and suggest a possible link between the gut-placental axis via Fusobacterium (Chen et al., 2020).

While there may be no definitive proof of the placental microbiome as of now, the above mentioned study suggests that a healthy, optimal microbiome may help in preventing placental pathophysiology in PE (Jin et al., 2022). If improved spiral artery remodeling was found due to the administration of certain species and SCFAs, it leaves the door open for the potential therapeutic effect that certain probiotics or metabolites may have (Jin et al., 2022).

## 4 The microbiome & the symptoms of pre-eclampsia

### 4.1 Inflammation

While inflammation is not necessarily a symptom of PE, it does play a huge role in the pathophysiology of the condition and the two are strongly associated together, as is immune response and the microbiome. It has been well defined that early life microbial colonization is key for normal development of the immune system (Takiishi et al., 2017). Early work examining the immune system in germ free mice demonstrated the importance of healthy microbes in not only the development, but also the priming of the immune system. These germ free mice have been found to have a mucosal immune system that is underdeveloped, fewer regulatory T cells, immature and impaired microglia, and were also not able to respond to immune challenges like normal, conventional mice (Peirce and Alviña, 2019). Separate studies have also demonstrated that host immune response can be altered by the presence of certain species in the gut, and these bacteria can push the development of various lymphocyte subtypes (Takiishi et al., 2017). This ranges from segmented filamentous bacteria stimulating the production of IL-17 and IL-22, to *Bacteroides fragilis* inducing Tregs in mice (Ivanov et al., 2009; Round et al., 2011).

The relationship between the gut microbiome and inflammation has been studied extensively in various other conditions from gastrointestinal to neurological. In inflammatory bowel disease, the composition of the human gut is altered, with low biodiversity, decreased Firmicutes and SCFA producers that would produce anti-inflammatory effects, and increased levels of Bacteroides, which can become pro-inflammatory (Sokol and Seksik, 2010; Morgan et al., 2012; Kolho et al., 2015). Patients suffering from multiple sclerosis, an autoimmune disorder affecting the central nervous system, have also displayed dysbiotic gut microbiomes, with the abundance of certain bacteria shifting drastically, including a decrease in Faecalibacterium and Bacteroides, and an increase in Streptococcus and Bifidobacterium (Mirza and Mao-Draayer, 2017). This dysfunctional relationship between the gut microbiome and inflammation also spans far beyond inflammatory and autoimmune disorders, also impacting the likes of mental disorders, such as major depressive disorder and generalized anxiety disorder (Jiang et al., 2015; Jiang H.Y. et al., 2018; Bastiaanssen et al., 2019).

However, the inflammation seen in PE, as previously mentioned, is heavily centered around the placenta (Magley and Hinson, 2022). Interestingly, in PE the placenta has been found to have a reduced amount of SCFAs in comparison to a normotensive pregnancies, correlating negatively to hypertension and proteinuria (Jin et al., 2022). By administering these SCFAs along with Akkermansia to PE rats, not only was the placental physiology improved, with increases in placental size and weight, but multiple inflammatory factors were also significantly downregulated (Jin et al., 2022). These include *Mif, Mcp1/Ccl2, Tlr4* and *Tnf* at both the mRNA and the protein levels (Jin et al., 2022). Furthermore, this intervention also reduced the antiangiogenic factor Eng, suggesting that this administration of the SCFAs may not only reduce placental inflammation but may improve blood vessel development (Jin et al., 2022). Separately, there are significantly elevated levels of LPS in the plasma of women with PE and the biosynthesis pathways in the microbiome were found to be slightly over-represented (Wang et al., 2019). Bacteroidetes, a known contributor to the LPS biosynthesis pathway, are increased in the PE microbiome, which, when produced, can enhance inflammation even more (Reuschel et al., 2019; Wang et al., 2019). This may also be true of other gram-negative bacteria that produce the endotoxin, potentially creating a cascade of more inflammation, more gut dysbiosis, and more effects on the host.

### 4.2 Hypertension

There is compelling evidence showing a link between the gut microbiome and hypertension. Studies have shown that germ-free mice have lower blood pressure, in comparison to those with a normal microbiome, and this may suggest that the microbiome may be able to regulate blood pressure (Moghadamrad et al., 2015). This may occur through the production of trimethylamine N-oxide by the microbiome, which is known to promote atherosclerosis, and increase the overall risk of various cardiovascular diseases including hypertension (Tang et al., 2013; Wang et al., 2015; Ge et al., 2020). Blood pressure changes in animals and non-pregnant individuals have been associated with a decline in overall microbial diversity and an altered Firmicutes:Bacteroidetes ratio (Yang et al., 2015). Furthermore, increased abundances of pathogenic genera were found, including Streptococcus and Klebsiella, whereas a decrease in *Faecalibacterium prausnitzii* and Roseburia were also found, both of these being SCFA producers (Yan et al., 2017). Administration of SCFAs has been shown to attenuate hypertension in preclinical murine models, highlighting their potential as a possible intervention (Bartolomaeus et al., 2019).

In relation to PE, genera such as Akkermansia display decreased abundance and has been negatively correlated with hypertension, and as previously described, hypertension can be alleviated in these animal models of PE following the administration of certain SCFAs and supplementing Akkermansia (Jin et al., 2022). Further animal studies have reproduced the same effects of SCFAs, but also noted the beneficial effects of other genera such as Lactobacillus and Bifidobacterium in alleviating genetic hypertension (Robles-Vera et al., 2020). PE has been strongly linked to numerous future cardiovascular events, including an increased risk of developing hypertension, stroke, heart failure, and coronary heart disease (Ahmed et al., 2014; Wu et al., 2017). Endothelial dysfunction, seen in PE, is strongly associated with cardiovascular disease (Widmer and Lerman, 2014). There is some preliminary evidence that the microbiome may contribute to endothelial dysfunction in some way. Studies have found that a high-fat, pro-inflammatory diet in mice led to endothelial dysfunction, with certain microbial metabolites potentially contributing to this (Toral et al., 2014; Kirichenko et al., 2020). It is possible that the dysbiotic microbiome may be exacerbating the severe cardiovascular complications seen in later life after the pregnancy.

### 4.3 Proteinuria

A definite link between microbial gut dysbiosis and proteinuria itself does not exist in the literature. However, there is thought that it may contribute in some way to the progression of the symptom (Kanbay et al., 2018). A considerable proportion of the studied conditions associated with proteinuria focuses on various kidney diseases, rather than PE. Across these diseases, one of the most obvious changes was a significant decrease in the beneficial, anti-inflammatory genera Bifidobacterium and Lactobacillus (Kanbay et al., 2018). Pro-inflammatory phylum such as Proteobacteria are found to be increased, along with genera such as Clostridium and Prevotella that can both have a positive impact by producing SCFAs. However, under certain conditions, these same bacteria can also produce toxins and LPS, which of course, can prove to be detrimental to the host (Kohn and Kung, 1995; Atarashi et al., 2011; Wong et al., 2014; Kanbay et al., 2018). Toxins produced by these bacteria have been linked to kidney damage (Tourountzis et al., 2022). In nephrotic syndrome, proteinuria is positively correlated with Thermoleopilia, Blautia, and Coriobacteriales, and negatively correlated with Alcaligenaceae and Betaproteobacteria (He et al., 2021). Mouse models of proteinuric disease, induced by Adriamycin, have also found changes in the composition of the gut microbiome, increased Odoribacter and decreased Rikenella and Turicibacter (Jiang Q et al., 2018).

In relation to PE, as previously mentioned, the gut microbiome plays a strong role in regulating blood pressure, which can contribute to proteinuria (Li et al., 2017; Kanbay et al., 2018). Various genera have been positively correlated with PE proteinuria, such as Fusobacterium and Veillonella, with negative correlations found for Akkermansia, Faecalibacterium, and Lachnospira (Chen et al., 2020). A recent study examined the effect of a faecal microbial transplant, from PE women and transferred to mice, demonstrated exacerbated proteinuria, amongst other symptoms in the mice, suggesting as previously noted, that the gut microbiome may contribute to the symptoms in some way (Chen et al., 2020).

## 5 Microbiome-targeted interventions

### 5.1 Probiotics, prebiotics, synbiotics, and postbiotics

Probiotics, which are beneficial bacteria, have been shown to modulate the gut microbiome, and promote an optimal environment and activity (Gerritsen et al., 2011). These beneficial bacteria are found in yogurt and cheese, along with being heavily supplemented in other foods such as cereals, and their use has been strongly recommended to aid in the treatment of conditions such as irritable bowel syndrome and cardiometabolic syndrome (Sniffen et al., 2018; Suez et al., 2019). Separately, prebiotics are typically carbohydrate-based substances that are non-digestible and are known to stimulate bacterial growth in the GI tract, can provide relief for constipation, and increase overall stool volume (Yadav et al., 2022). Together, prebiotics and probiotics are collectively referred to as synbiotics. Taken together, they have been shown to positively impact the health of the individual (Yadav et al., 2022). Many potential mechanisms have been put forward to explain how these bacteria produce their beneficial effects. These include the suppression of pathogens, improvement of GI barrier function, immunomodulation, and changing the signaling from the GI tract to the brain (Suez et al., 2019). As of recent, postbiotics have become something of interest. Defined as products derived from microorganisms that are no longer living and viable, it is thought that they exert their beneficial effects through anti-inflammatory, antioxidant, and immunomodulatory properties, along with enhancing gut barrier function (Żółkiewicz et al., 2020; Mosca et al., 2022; Vinderola et al., 2022). Potentially by alleviating inflammation, postbiotics could be useful in PE treatment. However, to our knowledge, this has not yet been studied. Despite this, they have shown to positively alleviate and improve symptoms in other conditions from irritable bowel syndrome to mild Alzheimer’s disease (Fujino et al., 2017; Andresen et al., 2020).

**Figure 2:**
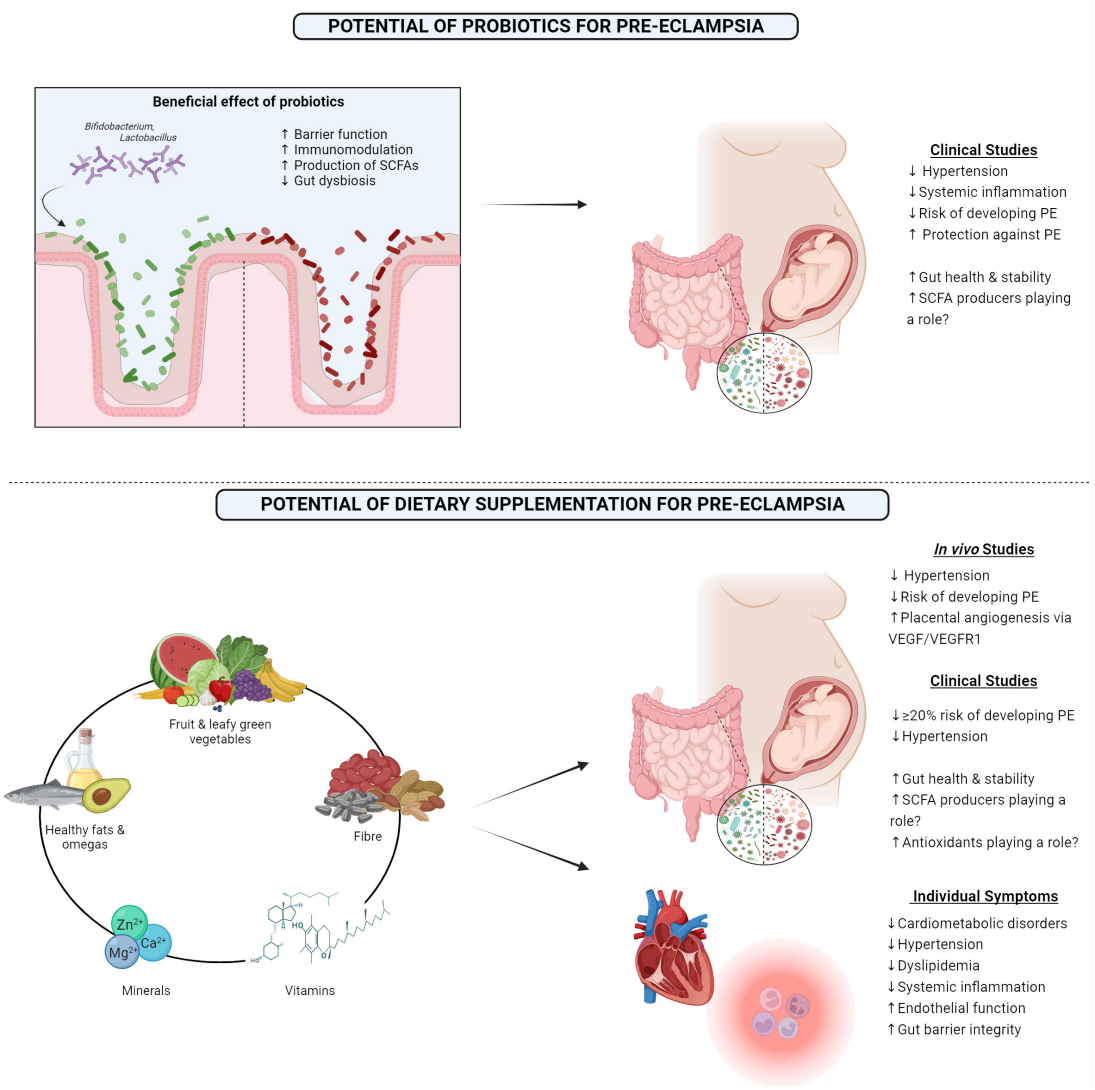
1) Showing the beneficial effects of probiotics and outcomes from clinical studies examining the outcome of probiotic consumption in pre-eclamptic women. 2) Highlighting the proposed optimal diet that may benefit pre-eclamptic women along with results from both *in vivo* and clinical studies, along with the clinical impact this diet has on individual symptoms which are associated with pre-eclampsia. PE, Pre-eclampsia; SCFA, Short-chain fatty acid; VEGF, Vascular endothelial growth factor; VEGFR1, Vascular endothelial growth factor receptor 1. Created with BioRender.com.

While few studies have examined the effect of synbiotics or postbiotics on PE, probiotics have been studied and have been suggested to benefit those women with PE by potentially positively modifying blood pressure, along with modifying both systemic and placental inflammation (Brantsæter et al., 2011) (**Figure 2**). A Norwegian study demonstrated that milk-based probiotics reduced the overall risk of developing PE, along with the risk of developing severe PE (Brantsæter et al., 2011). This has been supported by the findings in other studies, where probiotic milk was found to reduce the risk of developing PE. Interestingly, this was only seen when the probiotic was taken in the later stages of gestation (Nordqvist et al., 2018). Furthermore, a GWAS meta-analysis concluded that Bifidobacteria had a protective-style effect on PE (Li et al., 2022). These effects may also extend to other genera, Eubacterium, Lachnospiraceae, and Collinsella, all known SCFA producers (Li et al., 2022). Due to the strong evidence detailing the lack of SCFA-producing bacteria in the PE microbiome and the positive impact that supplementation had in animal models, probiotics known for producing SCFA, such as butyrate and proprionate, may potentially be a successful intervention in alleviating the maternal symptoms and lead to better outcomes for both the mother and the offspring.

### 5.2 Diet

Another potential way to modify the gut microbiome is through the diet to support the maternal gut microbiome, and subsequently help to improve the symptoms that are associated with PE. A large study performed in 2014 demonstrated that consumption of certain food items such as milk, chicken, pulses, and the nitrate-rich green leafy vegetables reduced the risk of developing PE (Agrawal, 2014). Other studies have noted the impressive potential of the Mediterranean diet for reducing the odds of PE, approximately a 20% reduction (Minhas et al., 2022). The mechanism behind this may potentially lie with enhanced placental vasculature due to improved endothelial function (Davis et al., 2017). Importantly, this reduced risk remains, even after adjusting for multiple sociodemographic factors, including ethnicity, despite certain ethnicities being at a greater risk of developing PE (Minhas et al., 2022). A high intake of fruit, vegetables, nuts, fish, legumes, and healthy fats have all been noted to lower the development risk (Brantsaeter et al., 2009; Endeshaw et al., 2015; Raghavan et al., 2019; Perry et al., 2022). Building on this, the Mediterranean diet has been proven to have many beneficial effects on the gut microbiome, such as enhanced microbial richness and increased levels of SCFAs, which coincided with a decrease in opportunistic pathogens, overall dysbiosis, and gut barrier leakiness (Merra et al., 2020).

In contrast to this, the Westernized diet typically consisting of low fiber, high-fat and processed foods such as red meat and white bread has been shown to increase the risk of PE (Ikem et al., 2019; Abbasi et al., 2021). In relation to the microbiome, this diet has routinely showed increased gut barrier permeability, decreased production of SCFAs, overall dysbiosis, along with an increase in pro-inflammatory bacteria that may produce toxins such as LPS (Malesza et al., 2021). Fiber has also shown to be a positive influence regarding risk, with those consuming high fiber (>24.3g per day) are over 50% less likely to develop PE in comparison to those on a low fiber diet (<13.1g per day) (Frederick et al., 2005). Importantly, adequate fiber consumption has been associated with lowering some of the key symptoms of PE, such as hypertension and dyslipidaemia, along with inflammation (Whelton et al., 2005; Qiu et al., 2008; Swann et al., 2020). However, there appears to be a fine balance regarding fiber consumption, as >30g of fiber per day has been associated with an increased risk of PE (Frederick et al., 2005).

Diets supplemented with omega-3 fatty acids and vitamin E have proved to have a positive impact in animal models by alleviating hypertension in rats with late-onset PE (Kasture et al., 2020). Placental angiogenesis was also found to be improved in late-onset PE rats post omega-3 and vitamin E supplementation, with increased levels of VEGF and its VEGFR1 receptor (Kasture et al., 2019). Omegas have also been found to reduce the risk of developing PE in the human population, however, these results have not been found to be consistent (Burchakov et al., 2017; Firouzabadi et al., 2022). Along with the positive effects of vitamin E in animal models, vitamin D with calcium supplementation has shown strong promise as a means of lowering the risk of PE (Khaing et al., 2017). The benefit of vitamin D has been found to be significantly greater if supplemented before 20 weeks gestation, but positive trends are still seen if the supplementation begins after this timepoint (Fogacci et al., 2020). A potential mechanism of this prevention could be attributed to the important role that vitamin D plays in vascular health and endothelial function (Cardús et al., 2006). Similar results have been found with other necessary dietary minerals, such as zinc, iron, magnesium, and phosphorous (Liu et al., 2023). The suggested mechanism behind this benefit may be due to the antioxidant properties of these compounds, reducing the volume of reactive oxygen species and free radicals (Liu et al., 2023). Folic acid has also shown to be beneficial in clinical studies, as overall risk of PE significantly decreased following appropriate supplementation (Liu et al., 2018). Mechanistically, this potentially may be due to folic acid lowering homocysteine levels, which when elevated is a risk factor in itself for PE, that can damage the vasculature of the placenta or promote apoptosis of trophoblast cells (Roberts and Cooper, 2001; Di Simone et al., 2004).

Due to the strong link between PE and inflammation, a successful intervention may lie in an anti-inflammatory-based diet. Poor, Western-style, high fat diets have been proven to drive gut dysbiosis, reduce SCFAs, and permeabilize the gut, all strongly intertwined with low-grade inflammation (Malesza et al., 2021). Over time, this coupled with a sedentary lifestyle can lead to chronic inflammation (Christ et al., 2019). Once again, the Mediterranean diet is known to lower inflammation, improve endothelial function, and reduce the risk of many serious health concerns, such as stroke and cardiovascular events (Schwingshackl and Hoffmann, 2014; Estruch et al., 2018). Unsaturated fatty acids and dietary fibers are two components of this diet that aid in the reduction of inflammation, and due to this, may be a possible intervention for those with PE (Christ et al., 2019). While the consumption of certain unsaturated fatty acids does seem to have a protective effect against PE, they do not seem to ameliorate hypertension (Bakouei et al., 2020). However, polyunsaturated fats have shown potential in modulating the inflammatory response seen in many diseases via the gut microbiome (Merra et al., 2020). Fiber has previously been proven to not only reduce the risk of developing PE, but can also reduce inflammation, dyslipidemia, and hypertension (Frederick et al., 2005; Whelton et al., 2005; Qiu et al., 2008; Perry et al., 2022). Hypertension, the key symptom of PE, may also be potentially managed through dietary interventions and modifications (Ozemek et al., 2018).

## 6 Conclusion

Recent work has strongly linked PE to the microbiome. However, whether a dysbiotic microbiome is the cause or simply just a consequence is still to be elucidated. Despite this gap in the knowledge that still remains, the microbiome in PE woman is considerably different in comparison to normotensive pregnancies. Building on this, the major symptoms of PE, hypertension, proteinuria, and associated inflammation, can also be separately traced back to a sub-optimal microbiome. Given this link, there is cautious optimism that microbiome-based interventions, such as a targeted diet rich in fiber, fruits, and vegetables, along with other essential nutrients or probiotic administration, particularly SCFA producers such as Bifidobacteria, as well as anti-inflammatory Lactobacillus species that may be of benefit in the reduction of symptoms or results of some symptoms. These potentially may improve the overall condition of PE and ideally provide a better environment for the developing fetus. More research must be done to fully understand the full potential of these interventions in a clinical setting.

## Author contributions

SO: Conceptualization, Funding acquisition, Supervision, Writing – original draft, Writing – review & editing. CD: Writing – original draft, Writing – review & editing. AB: Writing – review & editing, Writing – original draft. FM: Writing – review & editing, Supervision, Writing – original draft. GO: Funding acquisition, Writing – original draft, Writing – review & editing, Supervision. CM: Writing – original draft, Writing – review & editing, Supervision.
